# Pharmacological studies and pharmacokinetic modelling to support the development of interventions targeting ecological reservoirs of Lyme disease

**DOI:** 10.1038/s41598-024-63799-x

**Published:** 2024-06-12

**Authors:** Jérôme Pelletier, Catherine Bouchard, Cécile Aenishaenslin, Francis Beaudry, Nicholas H. Ogden, Patrick A. Leighton, Jean-Philippe Rocheleau

**Affiliations:** 1 Département de pathologie et microbiologie, Faculté de médecine vétérinaire, Université de Montréal, Saint-Hyacinthe, Québec Canada; 2 Groupe de recherche en épidémiologie des zoonoses et santé publique, Faculté de médecine vétérinaire, Université de Montréal, Saint-Hyacinthe, Québec Canada; 3 Centre de recherche en santé publique de l’Université de Montréal et du CIUSSS du Centre-Sud-de-l’Île-de-Montréal, Montréal, Québec Canada; 4 Public Health Risk Sciences Division, National Microbiology Laboratory, Public Health Agency of Canada, Saint-Hyacinthe, Québec Canada; 5 Département de biomédecine vétérinaire, Faculté de médecine vétérinaire, Université de Montréal, Saint-Hyacinthe, Québec Canada; 6 Centre de recherche sur le cerveau et l’apprentissage (CIRCA), Université de Montréal, Montréal, Québec Canada; 7 Département de santé animale, CÉGEP de Saint-Hyacinthe, Saint-Hyacinthe, Québec Canada

**Keywords:** Fluralaner, Pharmacology, Lyme disease, *Mus musculus*, *Peromyscus* spp., *Ixodes scapularis*, Reservoirs, Mathematical model, Ecological epidemiology, Infectious diseases, Bacterial infection, Ecology

## Abstract

The development of interventions targeting reservoirs of *Borrelia burgdorferi *sensu stricto with acaricide to reduce the density of infected ticks faces numerous challenges imposed by ecological and operational limits. In this study, the pharmacokinetics, efficacy and toxicology of fluralaner were investigated in *Mus musculus* and *Peromyscus leucopus* mice, the main reservoir of *B*. *burgdorferi* in North America. Fluralaner showed rapid distribution and elimination, leading to fast plasma concentration (C_p_) depletion in the first hours after administration followed by a slow elimination rate for several weeks, resulting in a long terminal half-life. Efficacy fell below 100% while C_p_ (± standard deviation) decreased from 196 ± 54 to 119 ± 62 ng/mL. These experimental results were then used in simulations of fluralaner treatment for a duration equivalent to the active period of *Ixodes scapularis* larvae and nymphs. Simulations showed that doses as low as 10 mg/kg have the potential to protect *P*. *leucopus* against infestation for a full *I*. *scapularis* active season if administered at least once every 7 days. This study shows that investigating the pharmacology of candidate acaricides in combination with pharmacokinetic simulations can provide important information to support the development of effective interventions targeting ecological reservoirs of Lyme disease. It therefore represents a critical step that may help surpass limits inherent to the development of these interventions.

## Introduction

*Borrelia burgdorferi *sensu lato*,* the bacterial agent of Lyme disease, is maintained in the environment by a complex transmission cycle involving populations of vertebrates and ticks of the genus *Ixodes*^1–4^. In eastern and central North America, *Peromyscus* mice and *Ixodes scapularis* ticks are the main reservoirs and vector for *B*. *burgdorferi *sensu stricto (hereafter *B*. *burgdorferi*), respectively^2^. In addition to their role as reservoir, *Peromyscus* mice are major hosts for *I*. *scapularis* larvae and nymphs^2,5,6^. Thus, they play a key role in the transmission of *B*. *burgdorferi* by amplifying the bacteria in populations of immature host-seeking *I*. *scapularis* ticks^5,6^. Therefore, treating *Peromyscus* mice with an acaricide that kills feeding larvae and nymphs could reduce the risk of Lyme disease transmission to humans, which is driven by the density of infected host-seeking *I*. *scapularis* ticks^7–10^.

Fluralaner is an acaricide in the isoxazoline family, used to kill ticks of various species infesting domestic animals^11^. Its primary mechanism of action is by inhibiting the opening of arthropods’ γ-aminobutyric acid-gated chloride channels^12–14^. Isoxazolines’ high specific affinity for arthropod receptors explains their safety at higher frequency and/or at many times the minimal effective dose when administered to mammals^13,15^. The efficacy of isoxazolines is linked to their concentration in animal plasma (C_p_)^16–18^. Fluralaner’s non-compartmental half-life (t_1/2_) in dogs after a single oral administration of a dose between 25 and 50 mg/kg is 12–14 days, meaning that it can kill > 90% of infesting *Ixodes* ticks on dogs for up to 3 months^16,17,19^. In dogs, fluralaner’s C_p_ goes up to 4000 ng/mL in the first hours following administration and remains over 100 ng/mL for up to 60 days^17^.

In a previous field study, the oral administration of fluralaner to *Peromyscus* mice through baits was associated with a 68–86% reduction of infestation with *I*. *scapularis* larvae and nymphs^20^. However, such a reduction may not result in a reduction of host-seeking infected ticks, as targeting reservoirs of *B*. *burgdorferi* may face limits related to ecological determinants of bacteria transmission, such as host community composition^20^. For example, if *B*. *burgdorferi* is maintained in the environment primarily by chipmunks, *Tamia striatus*, or short-tailed shrews, *Blarina brevicauda*, an intervention such as described in Pelletier et al. would not result in a significant reduction of Lyme disease risk^20^. Reservoir-targeted interventions must consider operational limits associated with treatment deployment modalities, i.e., number of baits, spatial extent of the treatment and quantity of acaricide require. These constraints can be influenced by the properties of the active ingredient. Efficacy, duration of action and safety all impose operational limits to an approach targeting small mammals with acaricides: the first two influence the dose to administer and at what frequency such a dose must be made available to small mammals, while the latter is related to possible toxic effects on targeted wildlife. Given the efforts needed to refill and distribute baits boxes over relatively large areas such as peri-urban nature parks or large backyards, minimizing the frequency at which bait boxes must be refilled would maximize operational feasibility. Overall, the optimal treatment strategy would maximize efficacy while minimizing frequency of application and toxicity.

In this study, we first investigated the pharmacokinetics (PK), efficacy and toxicology of fluralaner in three experiments. We then used these results to forecast and interpret outcomes of different treatment scenarios for *Peromyscus* mice. The underlying objective was to provide a comprehensive assessment of how features of an acaricide determine its applicability to and efficacy for Lyme disease risk reduction, using the case of fluralaner as an example.

## Results

### Pharmacokinetics

**Figure 1:**
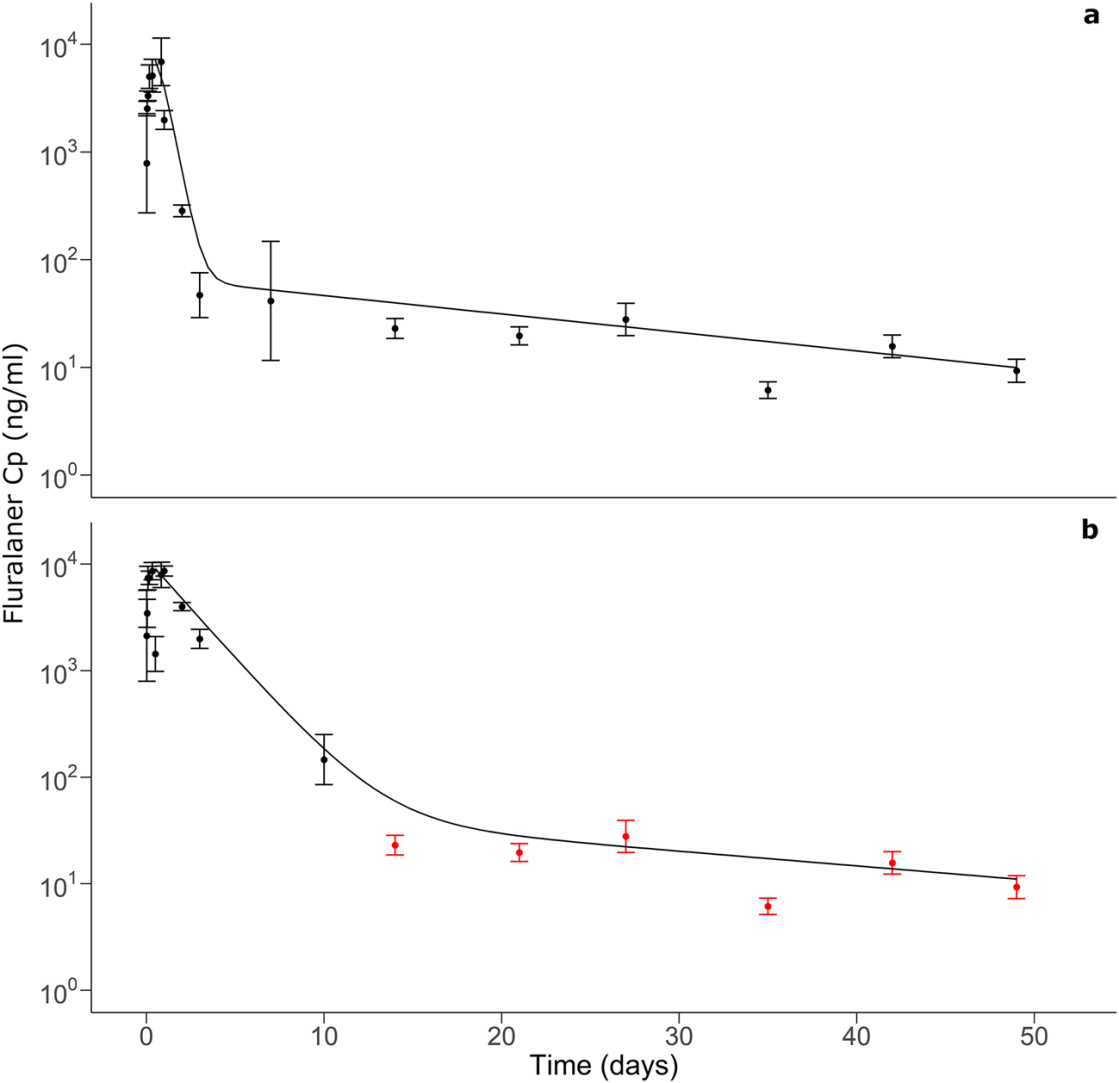
Pharmacokinetic profile of groups CD1-1 (**a**) and Pexx-1 (**b**). Dots are mean experimental C_p_ (± 1 SD) and lines are predicted values from compartment model fitted with experimental data. In b, the PK profile is incomplete past 10 days. The available blood volume of Pexx is small, therefore some samples had to be diluted with untreated *M*. *musculus* plasma to reach the proper volume to perform mass spectrometry analysis. To compute the micro-constants necessary to perform simulations, CD1 data (red dots) were used to complete the Pexx profile and fit a two-compartment model.

The PK of fluralaner was characterized using three groups of *M*. *musculus* (CD1, laboratory mouse lineage), and two groups of *P*. *leucopus* (Pexx, wild mouse lineage) (Table 1). The force-fed CD1-1 and Pexx-1 groups were used to characterize the complete PK profile of fluralaner, including the absorption, distribution and elimination phases. A two-compartment model had the best fit for the CD1 kinetics, while a one-compartment model was a better fit for the Pexx profile (Fig. 1; Supplementary Table S1). Each model’s goodness of fit with experimental data was assessed with the Akaike information criterion (AIC) and R^2^. The Pexx PK profile was incomplete past 10 days because fluralaner concentration was too low for quantification (Fig. 1b). Results showed fast absorption in both species, with absorption t_1/2_ (k_a_ − t_1/2_) and time to maximum C_p_ (T_max_) shorter, and maximum C_p_ (C_max_) higher in Pexx than in CD1 (Table 2). Fluralaner depletion during the distribution and elimination phases was slower in Pexx, with a longer distribution and elimination t_1/2_ (ɑ − t_1/2_) than the CD1 group (Table 2).

**Table 1:** Summary of experimental groups and their distribution between experiments.

Group	Dose (mg/kg)	Administration	Mice	Experiment
CD1-1	50	Force-fed	34	PK—complete profile—2 months
Pexx-1	50	Force-fed	37	PK—complete profile—2 months
CD1-2	50	Bait	10	PK—2 months
CD1-3	250	Bait	10	PK—2 months
Pexx-2	250	Bait	9	Efficacy and PK—2 months
Pexx-3	0	Bait	10	Efficacy
CD1-4	1000	Bait	10	Toxicology—euthanasia 7 days
CD1-5	0	NT	5	Toxicology—euthanasia 7 days
CD1-6	1000	Bait	10	Toxicology—euthanasia 30 days
CD1-7	0	NT	5	Toxicology—euthanasia 30 days

CD1 = *M. musculus* group; NT = no treatment; Pexx = *P. leucopus* group; PK = pharmacokinetics.

**Table 2:** Pharmacological parameters by experimental group.

Parameters	CD1-1^a^ Force-fed 50 mg/kg	CD1-2^a^ Bait 50 mg/kg	CD1-3^a^ Bait 250 mg/kg	Pexx-1^b^ Force-fed 50 mg/kg	Pexx-2^c^ Bait 250 mg/kg
C_max_ (ng/mL)	7357.0	NA	NA	10,070.4	NA
T_max_ (h)	9.4	NA	NA	4.5	NA
AUC_0→ t_ (d * ng/mL)	9242.7	9252.8	72,801.5	25,112.0	85,243.3
AUC_0→∞_ (d * ng/mL)	9495.5	9527.2	73,604.3	25,470.6	85,587.4
k_a_-t_1/2_ (h)	6.4	NA	NA	0.8	NA
α-t_1/2_ (h)	6.6	14.7	30.2	38.8	48.0
β-t_1/2_ (d)	17.6	18.2	61.1	NA	NA
MRT (d)	5.3	5.8	3.9	2.4	4.2

AUC_0→t_ = area under the curve from *t* = 0 to the last measurement time point; AUC_0→∞_ = area under the curve from *t* = 0 to infinity; α − t_1/2_ = distribution and elimination half-life; β − t_1/2_ = redistribution and elimination half-life; C_max_ = maximum plasma concentration; k_a_ − t_1/2_ = absorption half-life; MRT = mean resistance time; NA = not applicable; T_max_ = time to maximum plasma concentration.

^a^Computed from two-compartment models.

^b^Computed from a one-compartment model.

^c^Non-compartmental parameters.

**Figure 2:**
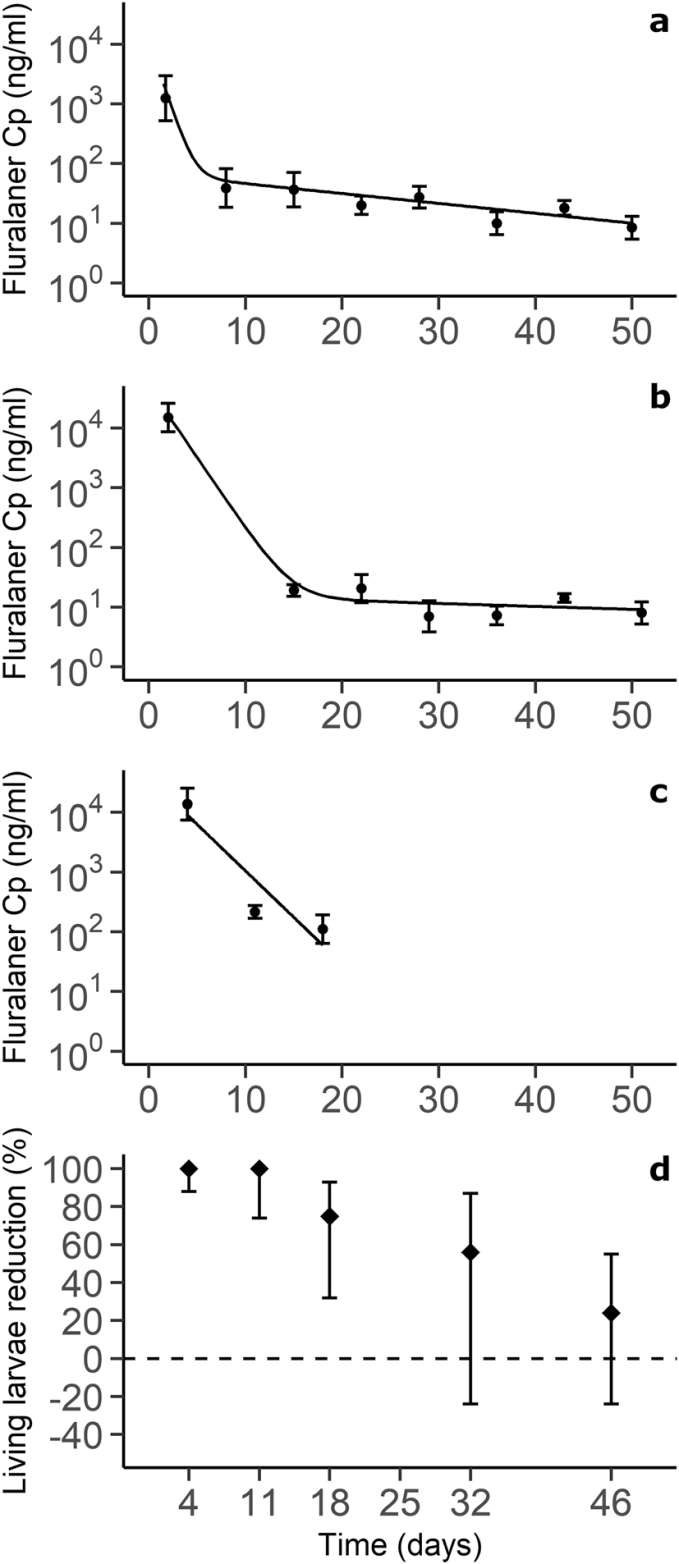
Pharmacokinetic profiles of groups CD1-2 (**a**), CD1-3 (**b**) and Pexx-2 (**c**), and the percent reduction in the number of counted attached living larvae in Pexx-2 (**d**). In panels (**a**–**c**), dots are experimental values (± 1 SD) and black lines are predicted values of two-compartment models fitted from experimental data. In c, not enough time points were available to perform compartmental analysis, therefore the black line is predicted values of a fitted log-linear regression. Dilution of samples with *M*. *musculus* plasma for mass spectrometry analysis brought fluralaner C_p_ below the limit of quantification. In panel d, the percent reduction in attached living larvae in the treated group compared with the control group is shown. The ratio of counts of attached living larvae was computed, with their 99% confidence intervals, with Poisson exact tests, and then percent reduction was calculated by subtracting the computed ratio from the null hypothesis value of 1. The 25-day efficacy assessment is not shown to ease visualization (see Supplementary Table S2).

The CD1-2, CD1-3, and Pexx-2 groups were used to characterize the PK profile of fluralaner over a two-month period after a single exposure to peanut butter baits containing fluralaner doses of 50 or 250 mg/kg (Table 1). The best fit for CD1 kinetics was a two-compartment model (Fig. 2a,b; Supplementary Table S1). In the Pexx-2 group, plasma volumes less than the 25 µL required for analysis resulted in not enough available time points to fit a compartment model with the PK profile of the group (Fig. 2c). The kinetic of group CD1-2, self-treatment through a bait, showed a longer ɑ-t_1/2_ than group CD1-1, force-fed. Group CD1-3 showed a longer redistribution and elimination t_1/2_ (β − t_1/2_) than group CD1-2 (Table 2). Every PK profile with β − t_1/2_ values showed a long C_p_ reduction phase lasting for weeks, in which fluralaner could be detected at low concentrations in blood (Table 2).

### Efficacy

The efficacy of fluralaner at killing *I*. *scapularis* larvae at chosen dosages was tested on two Pexx groups (Table 1). To assess treatment efficacy, we conducted experimental infestations at regular intervals. Percent reduction in attached living larvae was then calculated by comparing the ratio of attached living larvae between the Pexx-2 (treated) and Pexx-3 (placebo) groups. Data from experimental infestations are described in detail in Supplementary Table S2. On day 4 (CI99: 88–100) and 11 (CI99: 74–100), reduction in counted attached living larvae was 100% (Fig. 2d). Fluralaner efficacy began to decline between day 11 and day 18 (75%; CI99: 32–93) and showed no further significant reduction on day 25 (Supplementary Table S2). Between days 11 and 18, fluralaner C_p_ (± standard deviation [SD]) decreased from 196 ± 52 to 119 ± 62 ng/mL (Fig. 2c).

### Toxicology

This experiment was performed with four groups of CD1 mice. Groups CD1-4 and CD1-6, treated with 1000 mg/kg fluralaner baits, were compared with untreated groups CD1-5 and CD1-7 for clinical examination and for anatomical pathology (Table 1). Examination of the mice showed no difference between treated and control groups. There were no clinical signs of toxic effects after ingestion of fluralaner during follow-up of groups CD1-4 (7 days) and CD1-6 (30 days). After the follow-up period, mice were euthanized and examined for visible anatomical lesions (Supplementary File). Organs were weighed and samples were taken for a complete histopathological analysis on 3 individuals from each group. There was no significant difference in organ weight or relative weight (organ weight/mouse weight) between treated groups and their respective control groups (Supplementary Table S3). No visible or microscopic anatomical lesions were present in the filter organs of the treated groups.

Chemistry parameters of group CD1-4 and CD1-6 were compared with normal values from mice of the same age provided by Charles River Laboratories (Wilmington, MA, USA) (Supplementary Table S4). The analysis of hepatic and renal parameters did not increase, suggesting no toxic effect on these organs. One male mouse in group CD1-6 presented a high blood nitrogen urea (BUN = 35 mg/dl), but no other anomaly was identified. Both groups presented values for total proteins (TPR) slightly below normal values (Supplementary Table S4).

### Simulations

A PK model was built to simulate the movement of fluralaner between the gut, central and peripheral compartments based on three differential equations. Rates of transfer between compartments were computed from the CD1 and Pexx PK profiles (Fig. 1, Supplementary Table S5). A set of 100 different fluralaner doses (from 10 to 1000 mg/kg) and 30 different administration intervals between doses (from 7 to 210 days) were used to generate a total of 3000 treatment scenarios for a maximum treatment period of 210 days, which represents the larval and nymphal *I*. *scapularis* activity period in northeastern North America (April through October)^2^.

**Figure 3:**
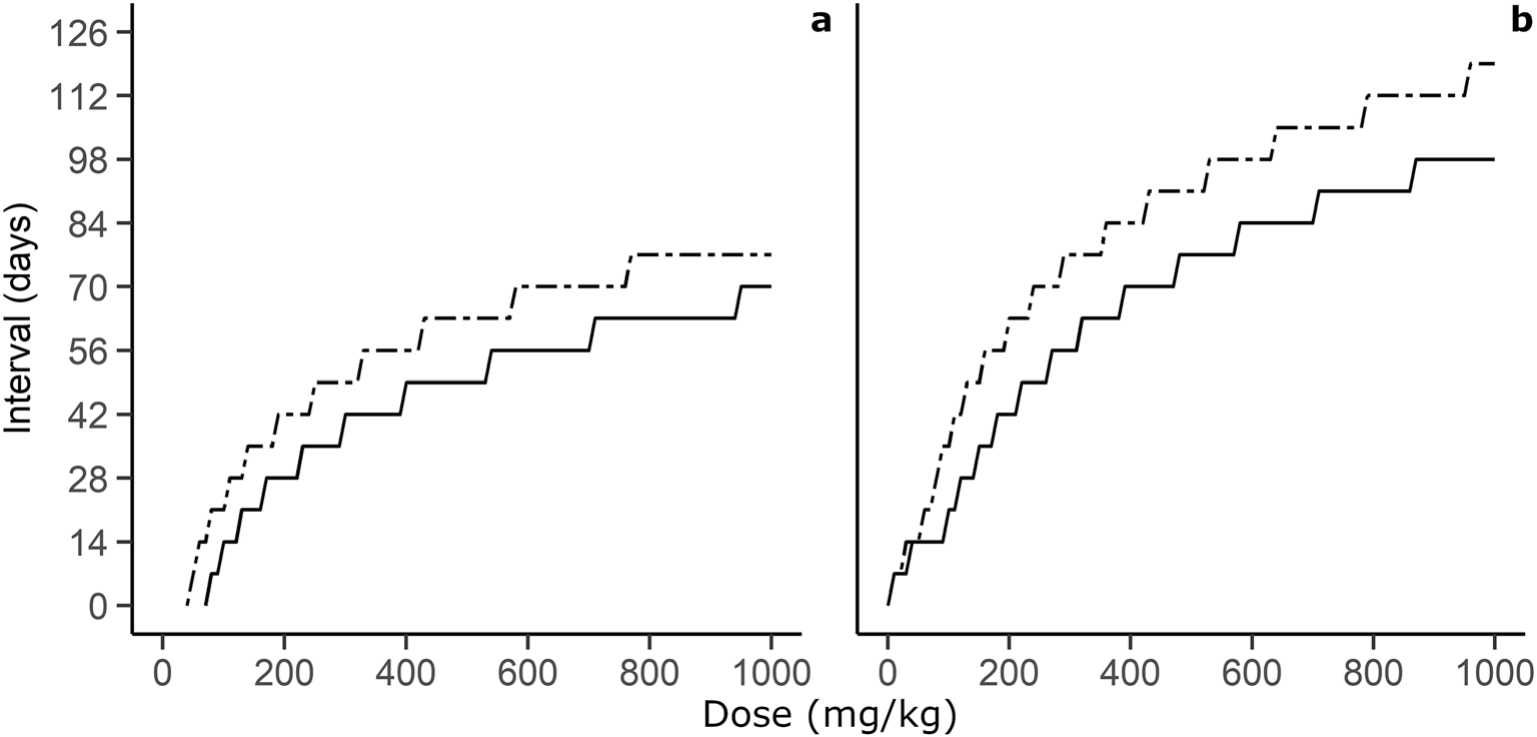
Maximum interval between doses that allows fluralaner C_p_ to stay over 196 ng/mL (solid line) or 119 ng/mL (dashed line) in CD1 (**a**) and Pexx mice (**b**). The C_p_ of 196 ng/mL and 119 ng/mL were associated with an efficacy of 100% and 75%, respectively.

**Figure 4:**
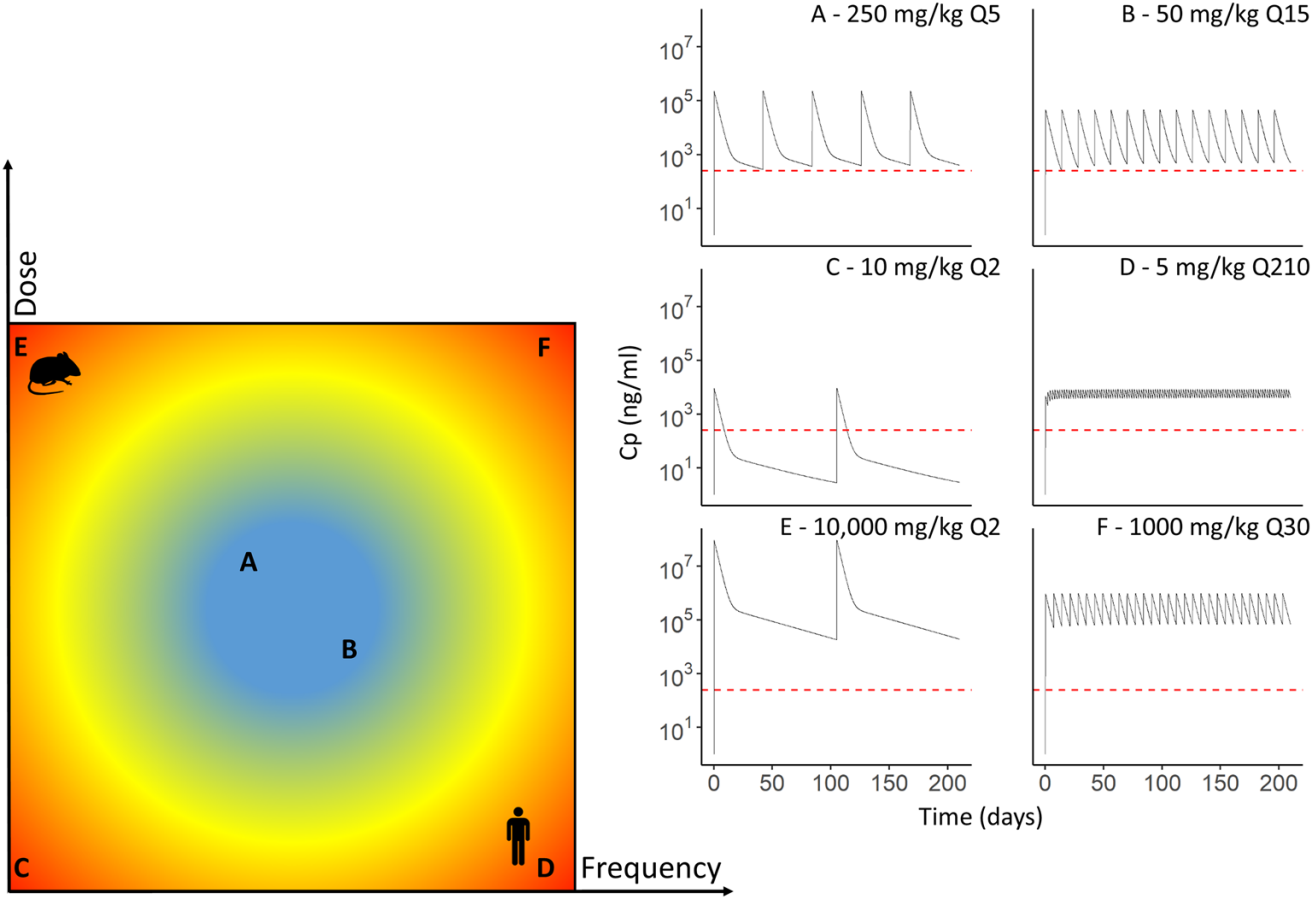
Schematic representation of different scenarios of treatment of Pexx mice with fluralaner. Scenarios (A, B, C, D, E and F) are distributed on the left-hand scheme according to the frequency (x axis) (Q = number of doses per 210 days) at what the treatment is rendered available dose (y axis). The blue color in the target indicates scenarios in which operational limits are weak, while red color indicates scenarios with high operational constraints. The C_p_ over time for each scenario is shown in the right-hand multi-panel plot. The red dashed line in the plots represents the 196 ng/mL threshold that provides a 100% efficacy against *I*. *scapularis* larvae according to experimental data. Scenarios A, B, C & F were selected among the scenarios tested in the study, while scenarios D & E were computed specifically to illustrate concepts. In the left-hand panel, the human icon beside scenario D indicate impracticability due to high dosing frequency. The need to replenish baits frequently increases costs and may reduce compliance. The mouse icon beside scenario E denotes impracticability due to the amount of fluralaner or bait required. With a large amount of fluralaner, baits could become toxic and unpalatable or simply too large to be fully consumed by mice.

Scenarios in which fluralaner C_p_ was > 196 ng/mL or > 119 ng/mL, the minimum concentrations with 100% and 75% efficacy respectively, for the 210-day period were extracted from the Pexx and CD1 model outputs. Maximum administration intervals between 2 doses that would allow C_p_ to stay over both thresholds were then computed and plotted (Fig. 3). Thirty-two percent (n = 960) and 22% (n = 660) of simulated scenarios resulted in 100% protection against infestation for 210 days for Pexx and CD1, respectively. Forty percent (Pexx, n = 1200) and 27% (CD1, n = 810) of scenarios could provide efficacy that would never go below 75%. The 960 scenarios providing 210-day protection against larval *I*. *scapularis* in Pexx included the administration of a 250 mg/kg bait every 42 days (a frequency of 5 administrations per season, scenario A in Fig. 4) or a 50 mg/kg bait every 14 days (a frequency of 15 administrations, scenario B in Fig. 4).

## Discussion

This study provides, to the best of our knowledge, the first extensive analysis of fluralaner pharmacology in *M*. *musculus* and *P*. *leucopus* mice. We used pharmacological experiments in a laboratory setting in combination with PK modelling to forecast the outcomes of different treatment scenarios on the efficacy of a treatment to kill immature *I*. *scapularis* ticks infesting *Peromyscus* spp. mice in the field. Our results suggest that the thorough study of the pharmacology of a candidate acaricide in a *B*. *burgdorferi* reservoir species, the approach described in this article, represents a critical step in the development of such interventions by providing essential data to guide the design of subsequent field experiments, thereby maximizing their likelihood of success. These results also highlight how pharmacological features of a specific acaricide may influence the development of interventions targeting *B*. *burgdorferi* reservoirs and impose operational limits.

The efficacy of isoxazolines is linked with their C_p_, which in turn depends on the dose administered and on the frequency at which this dose is made available^18^. These 2 parameters are critical to ensuring that animal C_p_ of the active ingredient remains in a therapeutic range. In this study, we aimed to apply this concept in the context of treatment of small mammal populations with acaricides. The treatment must be administered at a dose high enough to fully protect target animals against tick bites, and at a sufficient rate to ensure the animals maintain this protection long enough to maximize the effect on the transmission of *B*. *burgdorferi*. In this study, we first identified fluralaner pharmacological parameters that determine dosage and treatment effectiveness at killing infesting *I*. *scapularis* larvae. Then, the simulations provided different combinations of fluralaner doses and frequencies of administration that could protect susceptible *Peromyscus* mice against infestation by larval and nymphal *I*. *scapularis* for a complete season of activity. In the remaining scenarios, such as scenario C presented in Fig. 4, the C_p_ falls below the threshold of efficacy, which can mitigate the effect by reducing the fraction of immature *I*. *scapularis* ticks killed by the treatment. Although resistance to acaricides is unlikely in 3-host ticks since all other intermediate host species provide a refuge, such a scenario could create subtherapeutic conditions that may drive the development of drug resistance^21^.

While the model provides guidance regarding how the pharmacological properties of fluralaner in *Peromyscus* mice impose limits to interventions targeting *B*. *burgdorferi* reservoirs with acaricide, other operational limits must be highlighted. Some scenarios may be inapplicable despite resulting in a full 210-day coverage against larval infestation. Three of these are identified in Fig. 4. The first two scenarios (D and E) exceed the fluralaner dosing and frequency we deemed as practical in our PK simulations (Fig. 4), but were included to illustrate limits of this approach. Scenario D demonstrates limits related to human involvement (Fig. 4). Needing a human to refill baits every day may pose challenges regarding compliance to the treatment protocol and/or high cost if performed by workers. Scenario E highlights the potential negative effects if too much acaricide is administered (Fig. 4). High doses may result in toxic effects in target animals. Also, achieving such high C_p_ level in practical application would necessitate either using a too large amount of bait to be fully consumed by mice, or a highly concentrated formulation which may not be palatable (unpublished results). Finally, scenario F presented in Fig. 4 is part of the tested scenarios and combines both limits. It highlights the necessary balance between effectiveness and efficiency. Keeping mouse C_p_ high over the therapeutic threshold may represent a waste of compounds and of working time that may affect the cost-effectiveness of interventions. With a C_P_ steadily over 196 ng/mL and five bait box refills over the whole tick season, Scenario A, based on the dose tested in this study, seems to represent an applicable scenario. A similar approach to scenario B was used by Pelletier et al.^23^ in southern Québec between 2016 and 2019. Both scenarios A and B appear to be efficient treatment options. For precisely selecting the most appropriate treatment scenario among those deemed relevant according to the current study, factors influencing the way rodents interact with the baits and consumption rates, such as the bait’s fluralaner concentration and the time required for complete bait consumption in natural settings, should be further studied.

The results described in this study also highlight differences in fluralaner PK between mice and dogs. A faster C_p_ depletion in the first days is a major factor explaining the shorter duration of action in CD1 and Pexx mice. In CD1 mice, mean fluralaner C_p_ following a single force-fed oral dose of 50 mg/kg decreased below 100 ng/mL in less than 5 days, while Pexx mice mean C_p_ was still at or above 110 ng/mL 18 days after administration. Kilp et al. showed that, following a single dose of 50 mg/kg in dogs, mean C_p_ at or above 100 ng/mL was observed 56 days after administration of the treatment. The non-compartmental t_1/2_ of fluralaner in dogs was shown to be between 12 and 14 days^17^. In the present study, results suggest that fluralaner kinetics in mice fit better with a 2-compartment model, and analyses showed that t_1/2_ during the distribution and elimination phase is 6.6 and 38.8 h respectively for CD1 and Pexx mice. A hypothesis suggested to explain the rapid elimination in mice is higher blood flow to the liver, which is the main organ for the clearance of isoxazolines^16,17,22,23^. In our results, this phenomenon seems to be slower in Pexx mice. The explanations behind this observation are unclear, as little literature is available comparing these two species and investigating pharmacology in Pexx mice since they are not a common animal model used in the laboratory^24^. Exploring potential variation in hepatic hemodynamic and metabolism between these two species are potential research avenues. This difference between CD1 and Pexx mice may explain why doses as low as 10 mg/kg showed the potential to provide 210-day protection in the Pexx model whereas, in the CD1 model, no dose below 80 mg/kg showed such potential. It is plausible that using CD1 data after 10 days to complete the Pexx kinetic profile resulted in an underestimate of fluralaner C_p_ past that time point. However, most variation in fluralaner concentration occurs during the distribution phase, which is fully captured in the present study.

The C_p_ threshold of fluralaner efficacy reported in this study is similar to what is reported in the literature. Concurrently with the loss of 100% efficacy at killing *I*. *scapularis* larvae, i.e., between 11 and 18 days after treatment administration, fluralaner C_p_ decreased from 196 ng/mL (352 nmol/mL) to 119 ng/mL (213 nmol/mL). In comparison, afoxolaner is estimated to provide 90% efficacy at killing adult *Rhipicephalus sanguineus* ticks at a C_p_ of 110 ng/mL (301 nmol/mL) in dogs^18^. In their assessment of afoxolaner efficacy at killing adult *R*. *sanguineus* in dogs, Letendre et al.^18^ used a sigmoid maximum effect (E_max_) model. These models are used to describe the relationship between the concentration of a drug and its effect. Using such models to test the efficacy of acaricides in *Peromyscus* mice could help in determining efficacy thresholds and improve results used to interpret the outputs of the different scenarios. In this study, efficacy was tested only on larval *I*. *scapularis*; testing efficacy with nymphal ticks should be a further research avenue.

Providing baits with fluralaner doses as high as 1000 mg/kg did not result in toxic effects. This suggests that doses much higher than the recommended dose of 25 mg/kg in dogs or the 250 mg/kg tested in this study can be used with low probability of harming mice. An association with isoxazolines has been suspected in cases of neurological adverse events^25,26^. No such clinical signs were observed in the tested groups. However, the administration of fixed fluralaner doses through peanut butter baits consumed voluntarily could lead to disparities between the dose contained in the bait and the dose consumed by the animals, and the results provided by this study should be interpreted with this consideration in mind. This element of the study design was chosen to keep the conditions in the laboratory setting closer to those under which treatment is administered to small mammal populations in the field. In our view, this approach is the best way to represent the effects of treatment, in terms of efficacy and toxicity, when deployed in a field context.

The PK model outcomes may also be influenced by the fact that they were built with experimental data from force-fed groups. Fluralaner kinetics are influenced by administration with food, as reported in the literature and suggested by the PK results^27^. Administration with food is associated with higher bioavailability, which may result in a longer duration of action when fluralaner is administered through a peanut butter bait. Therefore, the models developed in this study could be further improved by incorporating bioavailability features.

Distributing baits containing acaricide in the environment, even if deemed safe for target species, poses potential risks to non-target organisms and to the environment. Treated mice could introduce the compound into the food chain by being consumed by predators and they can spread it through their feces. Isoxazolines are already present in the environment due to their widespread use in domestic animals and they are effective against various arthropod species beyond ticks^11,28^. Models like the one presented in this study could help minimize the overall environmental release of such compounds by optimizing treatment strategies and preventing over-application. Additionally, future models could incorporate features that explicitly assess the potential impact of these interventions on non-target species. Developing robust methods to assess the potential indirect impacts of such interventions will be a critical step prior to large-scale application of reservoir-targeted approaches using isoxazolines for infected tick control.

In summary, the results reported in this study highlight the importance of thorough pharmacological studies in reservoir species of *B*. *burgdorferi*, as they may influence further field studies and intervention design. In the context of the fluralaner treatment of *Peromyscus* mice, efficacy reduction in the first 2 weeks after the administration of a single oral dose impose operational limits that have a major influence on further development steps. To provide comprehensive guidance on the impact of those limits, we used an innovative transdisciplinary approach combining experimental data on fluralaner pharmacology with the development of a PK model to perform simulations of different treatment scenarios. Next steps in the development of the model could include the integration of economic data that could give guidance on the most cost-effective scenarios, such as Carrera-Pineyro et al. did with an intervention targeting small mammals with an anti-*B*. *burgdorferi* vaccine^29^. Interventions targeting reservoirs of *B*. *burgdorferi* must also take into account the ecological limits inherent to local drivers of bacteria transmission and of *I*. *scapularis* population amplification, such as host community composition ^30–33^. Eventually, models integrating acaricide PK, reservoir host population dynamics and various drivers of the *B*. *burgdorferi* endemic cycle may provide more complete assessment of effectiveness and more precise forecasted data of those interventions ^31,34–36^. We argue that these may become major tools to guide intervention development. This study is a first step in that direction for interventions targeting *B*. *burgdorferi* reservoirs with isoxazoline acaricides.

## Methods

### Animals

In this study, 84 CD1 mice (Charles River Laboratories), aged 3 months with a mean weight (± 1 SD) of 26.7 ± 2.8 g, and 58 Pexx mice (Peromyscus Genetic Stock Center, University of South Carolina, Columbia, SC, USA), aged from 6 to 12 months with a mean weight (± 1 SD) of 19.9 ± 3.1 g, were used. Upon arrival in their laboratory housing rooms, mice followed a minimal acclimatization period of 7 days prior to the study start. Each group was designed to have the same proportion of males and females. Mice were housed individually to ensure that only one mouse consumed the treatment and to avoid mutual grooming. They had continuous access to food (Charles River Rodents, Charles River Laboratories) and tap water for the duration of the study. Cages were enriched with shelters and nestlets to increase animal well-being. All mice were housed in the same room with a 12-h day/night light cycle; humidity was maintained between 50 and 70% and temperature between 22 and 25 °C. Experiments with CD1 and Pexx mice took place at different times to avoid contact between species. All animal experiments were performed in agreement with Canadian Council on Animal Care regulation, with the ethical approval of the institutional animal ethics committee of CÉGEP de Saint-Hyacinthe and in accordance with ARRIVE guidelines.

### Treatment administration and follow-up period

Oral treatment was administered by one of two routes: force-feeding (groups CD1-1 and Pexx-1) or baits (groups CD1-2, CD1-3, CD1-4, CD1-6, and Pexx-2). Force-feeding was given to experimental groups that were used to characterize PK during the first hour following administration. The force-feeding solution was composed of peanut oil and pure (> 98%) fluralaner powder (MedKoo Biosciences Inc., Morrisville, NC, USA); it was administered with a flexible cannula (Sigma-Aldrich, Saint-Louis, MO, USA) at a volume necessary to provide the targeted dose but not exceeding 10 mL/kg (Table 1). Baits were used with the aim of mimicking fluralaner PK, efficacy, or toxicology in a context as close as possible to that of treatment deployment in a natural environment. In this respect, baits were administered in the presence of their regular food to mimick food competition in the field. Baits were a mixture of peanut butter and a commercial formulation of fluralaner (Merck Animal Health, Madison, NJ, USA). The Pexx-3 group was a placebo control for the experimental group Pexx-2. As such, mice in this group received a bait made of pure peanut butter. Treatment consumption was assessed visually and by weighing baits within 48 h of administration. All baits were entirely consumed.

Mice were weighed periodically during the study. The general health status of the CD1-4, CD1-5 CD1-6 and CD1-7 groups were moreover followed up until pathology analysis.

### Infestation, larva count and anesthesia

For the Pexx-2 (n = 9) and Pexx-3 (n = 10) groups, experimental infestations took place at 2, 9, 16, 23, 30 and 44 days after treatment administration. Larvae used for infestation were provided by the Centers for Disease Control and Prevention for distribution by BEI Resources (Manassas, VA, USA) and by the National Microbiology Laboratory (Winnipeg, MB, Canada). To maximize larval attachment, mouse infestations were done under general anesthesia for 1 h with heater carpets as thermal support and with an injection of subcutaneous fluid (0.5 mL of NaCl 0.9%)^23^. During infestation, 20 unfed *I*. *scapularis* larvae were placed on the ears and fur of each mouse using fine-tipped forceps. Larvae were 3–6 months old and had typical host-seeking behaviours when applied on mice. At 48 h post-infestation, mice were visually inspected under anesthesia for a maximum of 5 min by observers blinded to the treatment, who followed a systematic procedure to count the number of attached larvae^24^. Subsequently, the attached larvae were removed with fine-tipped forceps and observed under a binocular microscope for a minimum of 1 min to characterize them as dead or alive according to the protocol developed by Pelletier et al.^23^.

All anesthesia performed in this study followed the same protocol. Animals were induced with a mixture of isoflurane and medical grade oxygen at a 5% concentration, and then maintained with an isoflurane concentration between 1 and 2%.

### Blood samples

Blood samples were taken by terminal intracardiac puncture under general anesthesia for the short-term PK (CD1-1 and Pexx-1). Three mice from each group were sampled at each short-term PK time point: 0.5 (n = 4 for group CD1-1), 1, 2, 4, 8, 20 (group Pexx-1 only), 24, 48 and 72 h after treatment administration. Repeated blood samples were taken via the lateral saphenous veins from the remaining animals in the CD1-1 and Pexx-1 groups to characterize fluralaner PK on a longer time scale. Five animals were sampled at a first time point (CD1-1 = 20 h and Pexx-1 = 12 h), then 5 animals were sampled in rotation every week for a maximum of 2 months.

The CD1-2, CD1-3 and Pexx-2 groups were used to characterize PK over a 2-month period Blood samples were taken from the lateral saphenous vein on 5 animals per group at 42 h (CD1-2), 48 h (CD1-3) and 96 h (Pexx-2) after treatment administration. Punctures from the lateral saphenous vein were then performed every week on subgroups of 4–5 mice in rotation. The last, intracardiac puncture, performed under anesthesia, was taken at 50, 51 and 45 days for the CD1-2, CD1-3 and Pexx-2 groups, respectively. For the Pexx-2 group, puncture was done on the day of the experimental infestations.

Blood samples from the CD1-4 and CD1-6 groups were taken through intracardiac puncture at the time they were sacrificed for pathology analysis. No blood samples were taken from groups CD1-5 and CD1-7.

### Determination of fluralaner blood concentration

To characterize fluralaner PK, blood was centrifuged at 1500×*g* for 10 min at 4 °C to extract the plasma. Plasma samples were analyzed by high-performance liquid chromatography mass spectrometry (HPLC–MS) to obtain fluralaner C_p_. For protein precipitation and fluralaner extraction, 100 µL (Pexx) or 200 µL (CD1) of internal standard solution (100 ng/mL of reserpine in methanol) was added to 25 or 50 µL of plasma samples, respectively. Obtaining the necessary volume of blood through the lateral saphenous vein from Pexx mice was difficult. Pexx mice are smaller than CD1 mice resulting in lower available plasma volumes. Therefore, if plasma volume was < 25 µL, samples were diluted with untreated *M*. *musculus* mouse plasma (Charles River Laboratories)^37^. The dilution factor was used after quantification to obtain the real C_p_. The sample was vortexed and left to stand for a period of 10 min, then centrifuged at 12,000×*g* for 10 min. The supernatant was transferred into an injection vial for HPLC–MS analysis after a standing time of 5 min. The HPLC system was a Vanquish FLEX UHPLC system (Thermo Fisher Scientific, San Jose, CA, USA) and the chromatography was achieved with a Thermo BioBasic Phenyl microbore column (Thermo Fisher Scientific) 50 × 1 mm, with a particle size of 5. The flow rate was fixed at 75 µL/min and 2 µL of samples were injected. A Q Exactive Orbitrap Plus mass spectrometer (Thermo Fisher Scientific) was interfaced with the Vanquish FLEX UHPLC system using a pneumatic-assisted, heated electrospray ion source. Chromatographic and mass spectrometry conditions and quantification procedures were the same as previously described by Pelletier et al.^23^. The observed precision and accuracy were < 15%.

### Clinical and anatomical pathology

Blood samples from groups CD1-4 and CD1-6 were transferred into dry tubes and sent to Biovet Inc. (Saint-Hyacinthe, QC, Canada) for biochemistry analysis. Subsequently, mouse carcasses were dissected according to a systematic procedure (Supplementary File). All samples were preserved in 10% formaldehyde at room temperature for a maximum of 3 months and were sent to the Laboratoire d’épidémiosurveillance animale du Québec (Saint-Hyacinthe, QC, Canada) for tissue preparation and histopathologic analysis.

### Data analysis

#### Compartmental analysis

Before analysis, the mean fluralaner C_p_ was computed for each group at every time point. One-compartment and two-compartment models were fitted, when sufficient data were available, on each group’s PK profile. PK parameters were computed from models that best fit experimental data. In cases fitting compartment models was not possible, PK parameters were estimated with non-compartmental analysis. PKsolver add-ons for Excel software (version 2108) was used for those analyses^38^.

#### Treatment efficacy

First, the ratios of the counts of attached living larvae (R_L_) between treated and control groups were computed, with their 99% confidence intervals, with Poisson exact tests using R software version 4.1.1^39^. This was done for each time point of treatment evaluation, i.e., at 4, 11, 18, 25, 32 and 46 days after treatment administration. Second, treatment efficacy was calculated by subtracting the computed ratio from the null hypothesis value of 1:

$${Efficacy}_{t} \left(\%\right)= 1-{R}_{t}$$

where *R*_*t*_ is *R*_*L*_ with their 99% confidence limits for an evaluation time point *t*. Under that formulation, the efficacy corresponds to the excess fraction of ticks killed because of the treatment 48 h after exposure of larvae to the treatment. It was assumed that the difference in killed ticks in the treatment group was due to the acaricide effect of fluralaner and not to any repulsive effect.

#### Pharmacokinetic model

Fluralaner micro-constants (*k*_*a*_, *k*_*12*_, *k*_*21*_ and *k*_*10*_) were computed from compartment models fitted in the PK experiment using PKsolver version 2108 (Supplementary Table S5)^38^. Micro-constants represent the rate of fluralaner molecule transfer between compartments. For the Pexx group, a two-compartment model was fitted by using CD1 experimental data past 10 days (Fig. 1). CD1 or Pexx micro-constants were used in 3 differential equations as transfer rates between 3 compartments: 2 pharmacological compartments (central = *C*_*p*_ and peripheral = *P*) and a gut compartment (*G*) used for treatment administration:

$$\frac{dG}{dt}=-{k}_{a}*{G}_{t}$$

$$\frac{d{C}_{p}}{dt}= {k}_{a}*{G}_{t}- {k}_{12}*{{C}_{p}}_{t}+{k}_{21}*{P}_{t}-{k}_{10}*{{C}_{P}}_{t}$$

$$\frac{dP}{dt}={k}_{12}*{{C}_{p}}_{t}- {k}_{21}*{P}_{t}$$

Simulations were performed by combining 100 fluralaner doses (from 10 to 1000 mg/kg in 10 mg/kg increments) introduced in the model through parameter G_t_ and 30 administration intervals (from 7 to 210 days in 7-day increments) for an equivalent duration of 210 days. The first dose was introduced in the model at *t* = 0 days then at each value of *t* days corresponding to the interval of administration. Simulations were performed using R version 4.1.1^39^. Differential equations were incorporated within a function, allowing for the inclusion of multiple drug administrations. The deSolve package's *ode* function and its *event* parameter facilitated the integration of event functions into the differential equation models^40^. A nested for-loop structure was employed to execute the function repeatedly. The first loop iterated through all administration intervals for the first dose, while the second loop encompassed the entire process for subsequent doses until completion. Data from the central compartment (parameter C_p_) represent fluralaner C_p_ (in ng/mL) associated with each of the 210-day treatment scenario. These data were extracted from the model output, and the maximum intervals allowing fluralaner C_p_ to remain above specific thresholds were then plotted against administered doses. The *ggplot2* and *cowplot* packages were used for data visualization^41,42^.

### Supplementary Information

Supplementary Tables.

Supplementary Information.

## Data Availability

Data available from Jérôme Pelletier on reasonable request.
