# Supplementary material for: Pharmacological studies and pharmacokinetic modelling to support the development of interventions targeting ecological reservoirs of Lyme disease
Source: Sci Rep. 2024 Jun 12;14:13537. doi: 10.1038/s41598-024-63799-x (PMC11169648; doi:10.1038/s41598-024-63799-x)
Supplement: Supplementary file 1 — Supplementary Tables. [file 41598_2024_63799_MOESM1_ESM.docx]

## Supplementary Tables

**Table S1.** Comparison of compartment model fit with experimental profiles.

| **Group** | **Treatment** | **One compartment** | | **Two compartments** | | **ΔAIC**^a^ |
| --- | --- | --- | --- | --- | --- | --- |
|  |  | **R^2^** | **AIC** | **R^2^** | **AIC** |  |
| CD1-1 | Force-fed - 50 mg/kg | 0.74 | 155.50 | 0.89 | 140.22 | -15.28 |
| Pexx-1 | Force-fed - 50 mg/kg | 0.80 | 10.58 | 0.76 | 13.49 | 2.91 |
| CD1-2 | Bait - 50 mg/kg | 0.99 | 71.82 | 0.99 | 53.01 | -18.81 |
| CD1-3 | Bait - 250 mg/kg | 0.99 | 53.55 | 0.99 | 46.91 | -6.64 |

Note: AIC = Akaike information criterion; CD1 = *M. musculus* group; Pexx = *P. leucopus* group

^a^One-compartment AIC value minus two-compartment AIC value; a negative value indicates a best fit of the two-compartment model; variation > 2 indicates a significantly better fit.

**Table S2.** Counts of *I. scapularis* larvae and ratios of larval count between groups Pexx-2 (fluralaner 250 mg/kg) and Pexx-3 (placebo), by time since treatment administration

| **Day since treatment**^a^ | **Parameters** | **Pexx-2 - 250 mg/kg** | | | **Pexx-3 - placebo** | | | **Ratio** [CI99]^f^ |
| --- | --- | --- | --- | --- | --- | --- | --- | --- |
|  |  | **Larval counts** | **n**^d^ | **Mean**^e^ [range] | **Larval counts** | **n** | **Mean** [range] |  |
| 4 | Attached^b^ | 31 | 9 | 3.4 [0 - 8] | 75 | 10 | 7.5 [2 - 13] | 0.46 [0.25 - 0.80] |
|  | Living (total)^c^ | 0 (26) | 9 | 0.0 [0 - 0] | 51 (70) | 10 | 5.1 [1 - 9] | 0.00 [0.00 - 0.12] |
| 11 | Attached | 18 | 9 | 2.0 [0 - 5] | 39 | 10 | 3.9 [1 - 5] | 0.51 [0.23 - 1.08] |
|  | Living (total)^c^ | 0 (15) | 9 | 0.0 [0 - 0] | 25 (38) | 10 | 2.5 [1 - 4] | 0.00 [0.00 - 0.26] |
| 18 | Attached | 20 | 9 | 2.2 [0 - 6] | 57 | 10 | 5.7 [3 - 9] | 0.39 [0.19 - 0.76] |
|  | Living (total)^c^ | 8 (20) | 9 | 0.9 [0 - 5] | 35 (56) | 10 | 3.5 [0 - 6] | 0.26 [0.07 - 0.68] |
| 25 | Attached | 37 | 9 | 4.1 [0 - 6] | 38 | 10 | 3.8 [1 - 7] | 1.08 [0.58 - 2.02] |
|  | Living (total)^c^ | 21 (33) | 9 | 2.1 [0 - 4] | 12 (38) | 10 | 1.2 [0 - 3] | 1.75 [0.74 - 5.74] |
| 32 | Attached | 17 | 8 | 2.1 [0 - 4] | 39 | 10 | 3.9 [0 - 7] | 0.54 [0.24 - 1.17] |
|  | Living (total)^c^ | 8 (15) | 7 | 1.1 [0 - 2] | 26 (39) | 10 | 2.6 [0 - 6] | 0.44 [0.13 - 1.24] |
| 46 | Attached | 50 | 7 | 7.1 [1 - 12] | 89 | 10 | 8.9 [6 - 11] | 0.80 [0.50 - 1.28] |
|  | Living (total)^c^ | 43 (50) | 7 | 6.1 [1 - 9] | 81 (89) | 10 | 8.1 [6 - 10] | 0.76 [0.45 - 1.24] |
| Total | Attached | 173 | 51 | 3.4 [0 - 12] | 337 | 60 | 5.6 [0 - 13] | 0.60 [0.47 - 0.77] |
|  | Living (total)^c^ | 80 (159) | 50 | 1.6 [0 - 10] | 230 (330) | 60 | 3.8 [0 - 11] | 0.42 [0.29 - 0.58] |

Note: Experimental infestations were performed 2, 9, 16, 23, 30 and 44 days after treatment administration with 20 larvae; larval counts were performed 48 hours after infestation.

^a^Number of days since treatment administration

^b^Number of attached larvae

^c^Number of attached larvae identified as alive; the number in brackets, when present, indicates the number of ticks analyzed to determine if dead or alive; the number is different from the total number of ticks counted because some larvae were damaged when removed from the mice.

^d^Number of mice contributing to the count

^e^Mean number of larvae per animal (total number of counted larvae divided by the number of animals contributing to the count)

^f^Ratio and confidence intervals were computed with Poisson exact tests.

| **Group** | **CD1-4** | **CD1-5** | **CD1-6** | **CD1-7** |
| --- | --- | --- | --- | --- |
|  | **1000 mg/kg - 7 days** | **Untreated - 7 days** | **1000 mg/kg - 30 days** | **Untreated - 30 days** |
| Animals (g) | 27.4 (4.4) | 30.0 (2.5) | 30.9 (6.1) | 29.0 (2.5) |
| Heart (mg) | 155 (30) | 180 (6) | 167 (29) | 158 (26) |
| Rel. (mg/g) | 5.7 (0.6) | 6.0 (1.5) | 5.6 (1.4) | 5.4 (0.6) |
| Kidney | 441 (120) | 585 (88) | 499 (165) | 378 (107) |
| Rel. (mg/g) | 15.9 (2.2) | 19.6 (3.0) | 15.9 (2.5) | 12.9 (2.5) |
| Liver (mg) | 1890 (533) | 1888 (117) | 1680 (498) | 1531 (238) |
| Rel. (mg/g) | 67.9 (9.7) | 63.4 (8.2) | 53.7 (9.1) | 52.8 (6.7) |
| Spleen (mg) | 118 (13) | 113 (36) | 122 (47) | 113 (12) |
| Rel. (mg/g) | 4.4 (3.9, 4.9) | 3.7 (1.1) | 4.0 (1.2) | 3.9 (0.6) |

**Table S3.** Mean weights of animals and principal organs (SD) for each group included in the toxicology experiment

Rel. = relative weight (organ weight/mouse weight)

Note: Differences between groups were tested with the Mann-Whitney Wilcoxon test.

| **Parameters** (units) | **Sex** | **Normal values**^a^ | **CD1-4** | **CD1-6** |
| --- | --- | --- | --- | --- |
|  |  |  | 1000 mg/kg - 7 days | 1000 mg/kg - 30 days |
| ALT (U/L) | F | 63 (40, 170) | 25 (22, 27) | 24 (19, 29) |
|  | M | 60 (41, 131) | 32 (19, 51) | 26 (23, 52) |
| AST (U/L) | F | 154 (67, 381) | 85 (70, 96) | 118 (85, 138) |
|  | M | 135 (55, 352) | 164 (73, 352) | 79 (52, 184) |
| BUN (mg/dL) | F | 20 (7, 31) | 24 (21, 25) | 23 (17, 26) |
|  | M | 18 (7, 26) | 25 (24, 26) | 24 (18, 35) |
| GGT (U/L) | F & M | 18 | < 5 | < 5 |
| Creatinine (μmol/L) | F & M | 0.4 (0.2, 0.5) | < 0.27 | < 0.27 |
| GLU (mg/dL) | F | 193 (85, 281) | 182 (169, 191) | 145 (110, 169) |
|  | M | 206 (129, 329) | 184 (155, 209) | 148 (124, 184) |
| TPR (g/dL) | F | 6.0 (4.9, 7.3) | 4.7 (4.6, 4.8) | 4.9 (4.6, 5.2) |
|  | M | 6.1 (4.8, 8.7) | 4.4 (4.2, 4.6) | 4.5 (4.3, 4.7) |

**Table S4.** Comparison between mean values (range) of biochemistry parameters of groups CD1-4 and CD1-6 and mean normal values (CI95) of CD1 *M*. *musculus* mice of the same age

ALT = Alanine aminotransferase, AST = Aspartate aminotransferase, BUN = Blood urea nitrogen, F = Female, GGT = Gamma-glutamyl transferase, GLU = Glucose, M = Male, TPR = Total protein refractometer.

^a^Data provided by Charles River Laboratories.

**Table S5.** Micro-constants computed from compartment model fitted to pharmacokinetic profiles of CD1-1 and Pexx-1

| **Micro-constant** | **Effect** | **CD1-1** | **Pexx-1**^a^ |
| --- | --- | --- | --- |
| k_a_ (1/d) | G 🡪 C_p_ | 2.61 | 21.06 |
| k_12_ (1/d) | C_p_ 🡪 P | 0.44 | 0.02 |
| k_21_ (1/d) | P 🡪 C_p_ | 0.05 | 0.03 |
| k_10_ (1/d) | C_p_ 🡪 E | 2.06 | 0.41 |

C_p_ = central compartment, E = eliminated, G = gut compartment, P = peripheral compartment.

^a^To calculate *P*. *leucopus* micro-constants, a two-compartment model was fitted on data from the Pexx-1 group completed past 10 days with data from the CD1-1 group.
