# Supplementary material for: Pharmacological studies and pharmacokinetic modelling to support the development of interventions targeting ecological reservoirs of Lyme disease
Source: Sci Rep. 2024 Jun 12;14:13537. doi: 10.1038/s41598-024-63799-x (PMC11169648; doi:10.1038/s41598-024-63799-x)
Supplement: Supplementary file 2 — Supplementary Information. [file 41598_2024_63799_MOESM2_ESM.docx]

**Necropsy procedure**

Following euthanasia, mice from group CD1-4, CD1-5, CD1-6 and CD1-7 were necropsied according to the following systematic procedure:

1. Mice were weighted before dissection.
2. Skin was removed.
3. Salivary glands and mandibular lymph nodes were extracted.
4. Abdominal cavity was opened:
5. Hearth, liver, the 2 kidneys and spleen were extracted and weighted.
6. Reproductive tract, adrenal gland and digestive organs were extracted.
7. Formaldehyde 10% were injected in gut lumen.
8. Thoracic cavity was opened.
9. Respiratory tract was extracted with tongue, larynx and thyroid gland.
10. Formaldehyde 10% were injected in lungs to avoided atelectasis.
11. Mice head and right pelvic members were reserved for brain, nerves and muscles analyses.

During the procedure and before organs extraction, macroscopic observations were noted. Organs and body parts were preserved in designated container for each individual with formaldehyde 10%.
